# Future Prospects, Approaches, and the Government’s Role in the Development of a Hepatitis C Virus Vaccine

**DOI:** 10.3390/pathogens13010038

**Published:** 2023-12-31

**Authors:** Ashraf A. Tabll, Sayed S. Sohrab, Ahmed A. Ali, Ana Petrovic, Sabina Steiner Srdarevic, Stjepan Siber, Marija Glasnovic, Robert Smolic, Martina Smolic

**Affiliations:** 1 Microbial Biotechnology Department, Biotechnology Research Institute, National Research Centre, Cairo 12622, Egypt; 2 Egypt Centre for Research and Regenerative Medicine (ECRRM), Cairo 11517, Egypt; 3 Special Infectious Agents Unit, King Fahd Medical Research Center, King Abdulaziz University, Jeddah 21589, Saudi Arabia; ssohrab@kau.edu.sa; 4 Department of Medical Laboratory Technology, Faculty of Applied Medical Sciences, King Abdulaziz University, Jeddah 21589, Saudi Arabia; 5 Molecular Biology Department, Biotechnology Research Institute, National Research Centre, Cairo 12622, Egypt; aa.attia@nrc.sci.eg; 6 Faculty of Dental Medicine and Health Osijek, Josip Juraj Strossmayer University of Osijek, 31000 Osijek, Croatia; anapetrovic@fdmz.hr (A.P.); ssteiner@fdmz.hr (S.S.S.); ssiber@fdmz.hr (S.S.); marija.glasnovic@mefos.hr (M.G.); rsmolic@fdmz.hr (R.S.)

**Keywords:** hepatitis C virus, vaccine, SARS-CoV-2, COVID-19, government agencies, prevalence

## Abstract

Developing a safe and effective vaccine against the hepatitis C virus (HCV) remains a top priority for global health. Despite recent advances in antiviral therapies, the high cost and limited accessibility of these treatments impede their widespread application, particularly in resource-limited settings. Therefore, the development of the HCV vaccine remains a necessity. This review article analyzes the current technologies, future prospects, strategies, HCV genomic targets, and the governmental role in HCV vaccine development. We discuss the current epidemiological landscape of HCV infection and the potential of HCV structural and non-structural protein antigens as vaccine targets. In addition, the involvement of government agencies and policymakers in supporting and facilitating the development of HCV vaccines is emphasized. We explore how vaccine development regulatory channels and frameworks affect research goals, funding, and public health policy. The significance of international and public-private partnerships in accelerating the development of an HCV vaccine is examined. Finally, the future directions for developing an HCV vaccine are discussed. In conclusion, the review highlights the urgent need for a preventive vaccine to fight the global HCV disease and the significance of collaborative efforts between scientists, politicians, and public health organizations to reach this important public health goal.

## 1. Global and Egypt Prevalence of HCV

HCV is a global health problem, with an estimated 58 million people living with the disease, which accounts for about 1% of the world’s population [1]. In 2015, globally, there were 237 cases of HCV per 100,000 people and an estimated 1.75 million new infections of HCV. HCV has been classified into 8 genotypes and 93 subtypes (https://ictv.global/sg_wiki/flaviviridae/hepacivirus (accessed on 29 December 2023) [2]. HCV genotypes 1 (44%), 3 (25%), and 4 (25%) are the most common genotypes in the world. In 20–30 years, about 10–20% of people who have had HCV for a long time are likely to develop problems such as cirrhosis, end-stage liver disease, or hepatocellular carcinoma [3,4]. Only 20% of hepatitis C-infected patients are actually diagnosed, and 15% of those have been treated. To meet the WHO 2030 elimination goals, everyone should get affordable point-of-care diagnostics and pan-genotypic direct-acting antiviral therapy, as reported [1]. Egypt used to have the highest HCV infection rate. Two probability-based studies conducted in 2008 and 2015 suggested a decline in HCV antibody prevalence from 14.7% to 10%, respectively [3,5,6,7]. The most recent prevalence data were gathered from Egypt’s national initiative for HCV screening and treatment. From 2018 to 2019, Waked I [8] found an overall prevalence rate of 4.6% of HCV antibody-positive individuals and 3.5% of individuals with HCV viremia within the screened cohort (consisting of about 50 million Egyptians). This lowering trend in anti-HCV prevalence among Egyptian general populations may be linked to advancements in blood screening and infection control methods over the last two decades, as well as the national treatment campaign and its effectiveness as a preventive tool [9]. However, recent findings by Khattab et al. [10] revealed the persistence of HCV RNA in some HCV patients who achieved a sustained virologic response (SVR) after DAA treatment.

## 2. Structural and Non-Structural Protein Antigens as Targets for HCV Vaccine

**Figure 1:**
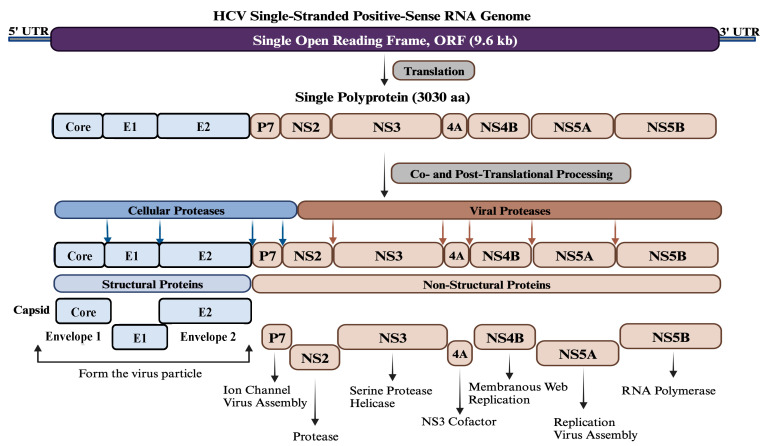
Genome organization of HCV. HCV contains a single-stranded positive-sense RNA genome of about 9.6 kb in size. The genome has a single open reading frame (ORF) flanked by two untranslated regions (UTR) at both ends. The ORF encodes a single polyprotein of approximately 3030 amino acids, which is processed co- and post-translationally by various cellular and viral proteases into three structural proteins (Core, E1, and E2) at the N-terminus and seven non-structural proteins (P7, NS2, NS3, NS4A, NS4B, NS5A, and NS5B) at the C-terminus. Structural proteins oligomerize and self-assemble to form the viral particle, while non-structural proteins are involved in viral assembly and genome replication.

The HCV is a spherical particle and consists of a single-stranded positive-sense RNA molecule enclosed within a capsid composed of multiple subunits of the core protein. The RNA genome and core protein assemble to form the nucleocapsid. Surrounding the nucleocapsid is an envelope composed of two viral glycoproteins, E1 and E2, embedded in a lipid bilayer derived from host membranes [11]. The HCV genome size is approximately 9.6 kb and contains a single open reading frame (ORF) encoding a single polyprotein, which is processed by cellular and viral proteases into structural (Core, E1, and E2) and non-structural proteins (P7, NS2, NS3, NS4A, NS4B, NS5A, and NS5B) (Figure 1). Given the current status of disease burden and available therapeutics, a prophylactic vaccine against HCV is urgently required. Globally, various therapeutic strategies are being used for HCV infections, such as DAAs, host-targeting agents (HTAs), micro-RNAs, nanomedicine, and immunotherapeutic approaches. In parallel, HCV vaccines’ candidates are being designed, developed, and experimentally evaluated using different strategies and platforms, including bioinformatics approaches targeting different regions of the viral genome. The continuous advancement of global research has provided significant and valuable insights into the structural and non-structural proteins of HCV to identify the main targets and epitopes for effective HCV vaccine design and development. While the Core, E1, and E2 proteins have been predominantly utilized as vaccine targets compared to the non-structural proteins, ongoing efforts aim to explore the full potential of these proteins in HCV vaccine design [12,13,14,15,16,17,18,19,20,21,22,23,24,25].

## 3. HCV Vaccines Targets and Technologies

Various strategies are being employed in the development of HCV vaccines to trigger immune responses. These strategies include the use of neutralizing antibodies (nAbs) that target exclusively the envelope E1/E2 proteins, the induction of T cell responses against the core protein or non-structural proteins (either individually or in combinations), the utilization of peptides (truncated isoforms or fragmented ones) and the development of multiepitope proteins using different technologies such as RNA interference and nanotechnology. Notably, the envelope glycoproteins (E1/E2) have been extensively utilized in HCV vaccine development. These envelope proteins are well-characterized proteins located on the surface of HCV virion and are known to be highly glycosylated transmembrane proteins (5 glycans on E1 and up to 11 on E2). The generation of neutralizing antibodies (nAbs) directed against E1, E2, and other viral proteins can confer clearance and protection against HCV infection [19,23,25,26,27].

### 3.1. Structural Proteins

#### The Envelope Glycoprotein 1 (E1)

**Figure 2:**
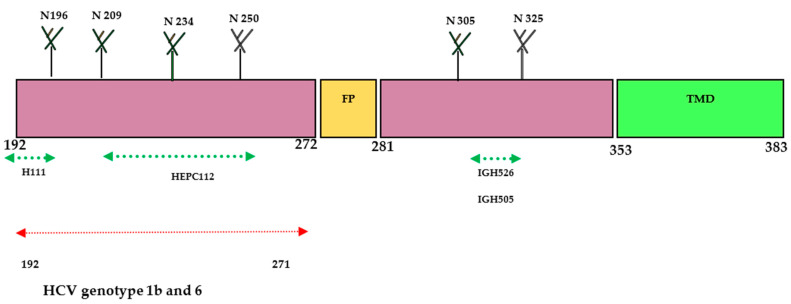
Schematic diagram of HCV- E1 envelope protein. E1 contains N-terminal domain (NTD, 192–383). The size of the E1 protein is 190 aa and is divided into ectodomain (160aa) and transmembrane domain (TMD) (30aa). Four N-glycosylation sites (N196, N209, N234, and N305) are conserved in all genotypes; N250 is found in genotypes 1b and 6, but N325 is absent when a proline residue is present at Asn-X-Ser/Thr. The crystal structure of the N-terminal domain of E1 considered individually was determined (PDB:4UOI), as well as the region 314–324 (PDB: 4N0Y) by co-crystallization with the human antibody IGH526. Antibody binding sites: aa 192–202 for the human monoclonal antibody (mAb) H-111, aa 215–299 for the human mAb HEPC112, and aa 313–324 for the human mAbs IGH505 and IGH526.

The E1 protein has been extensively studied by various research groups and is identified as one of the most important targets for developing nAbs against HCV. While the size of the E1 protein is small, it exhibits better conservation compared to E2 and has been less characterized in terms of its crystal structure. Notably, E1 protein contains antigenic sites in the fragmented residues 192–271 and 314–324 on mature virion. Based on the recently characterized structure of the E1 protein, it has been reported that the E1 protein significantly contributes to the fusion step by using the putative fusion peptide (FP) and transmembrane domain (TMD) (Figure 2). The heterodimer structure of the full-length E1 protein has generated novel elements for the development of an HCV vaccine. The envelop glycoproteins E1 (aa192–383) are located at the N-terminus of the envelope-spanning region of the polyprotein (aa192–746). So far, only two immunogenic regions have been identified for neutralizing antibodies (nAbs) at the N terminus (aa192–207) and C terminus (aa313–328) of the E1 protein [14,22,28].

The E1 protein participates in the viral entry and fusion step during HCV infection through its interaction with cell membrane receptors known as claudin (CLDN)-1, CLDN-6, and CD36 [22,29]. In 2014, a research group successfully crystallized the N-terminal domain of the E1 protein from genotype 1 (H77 strain) under low-pH conditions [28]. Interestingly, the crystallized protein exhibited a smaller size and resembled phosphatidylcholine transfer protein rather than the fusion proteins of flavivirus. However, another research group reported conflicting findings, suggesting that the N-terminal domain of the E1 protein differed from the structure determined by the earlier group [28,30]. Their research confirmed that E2 protein is needed for the proper folding of E1 protein. Further investigations revealed that the E1 protein can form a trimeric structure through the GxxxG motif situated at its transmembrane domain, which facilitates the interaction between the E1 and E2 proteins [30]. A human monoclonal antibody (mAb) H-111 was generated against Gt 1b HCV, targeting the linear epitope within the amino acid range of 192–202. Although a high titer was observed, it displayed weak neutralizing activity against HCV pseudo particles. Additionally, two other mAbs (IGH505 and IGH526) were identified to target the region between aa 313 and 328. These antibodies demonstrated strong neutralization activity against HCVpp of genotypes 1a and 2a in cell culture. Another study conducted by a different research group showed that a broadly neutralizing antibody called HEPC112, developed against antigenic site (AS) 112 spanning amino acids 215–299 of the E1 protein, was able to neutralize seven strains of HCV [22,31]. These findings highlight the structural and immunological complexity of the E1 protein and underscore the potential of specific antibodies to target various epitopes within this protein. Further research and characterization of the E1 protein hold promise for advancing our understanding of HCV infection and facilitating the design of effective preventive strategies against this viral disease.

### 3.2. The Envelope Glycoprotein 2 (E2)

**Figure 3:**
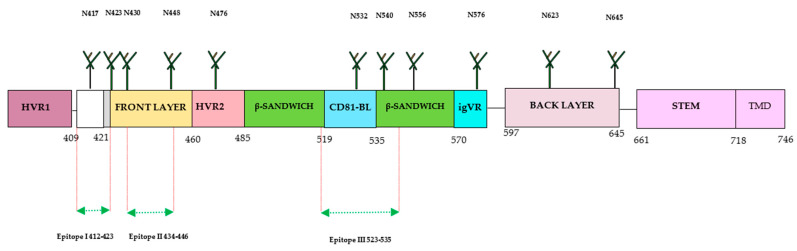
Structure of HCV E2 protein. The E2 consists of 360 aa, which is divided into 30 aa for the transmembrane domain (TMD), and consists of 3 variable regions (HVR1, HVR2, and igVR), including front layer, a back layer, CD81-binding loop (CD81bl), as well as STEM region, TMD regions start at 718 and end at 746, and 11 N-glycosylation sites. The epitope 1 (aa412–423) can adopt three conformations: beta hairpin, semi-open, and open. The epitope II (aa434–446) was co-crystallized with human monoclonal antibodies (mAbs) (H-84-27) and HC-84-1. The crystal structure of epitope III (523–535) was also obtained by co-crystallization with the mouse mAb DAO5 and targeted by the mAbs 1:7 and A8.

The E2 protein has emerged as a prominent target for the development of HCV-specific neutralizing antibodies (nAbs) compared with other proteins, [13,15,22]. The full-length structure of the E2 ectodomain was successfully resolved by several research groups, providing significant structural insights for the development of the HCV vaccine. The E2 protein consists of hypervariable regions (HVR1 and HVR2), an intergenotypic variable region (igVR), β-sandwich (aa492–566) flanked by a front (aa424–459) and a back layer (aa597–645), and a STEM and transmembrane domain (TMD) (Figure 3). The E2 protein contains three variable regions that facilitate viral escape from immune responses. The E2 (aa384–746) is located at the C-terminus of the envelope region of the HCV polyprotein (aa192–746). 

**Figure 4:**
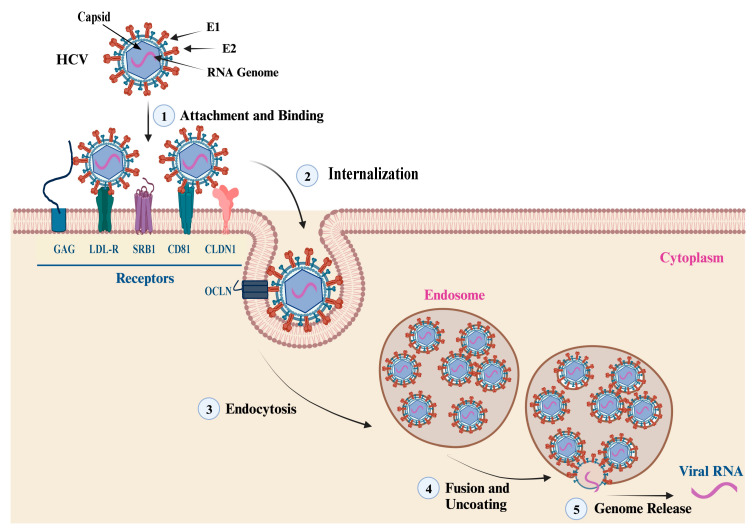
HCV Attachment and Cell Entry. This figure summarizes the main steps of HCV particle cell entry and genome release. (1, 2) The attachment, binding, and internalization are facilitated by the interaction of the viral envelope glycoproteins (E1 and E2) with cell membrane receptors, co-receptors, and host factors. The main cell receptors and factors that mediate the virus attachment and cell entry include glycosaminoglycan (GAG), low-density lipoprotein (LDL-R), scavenger receptor class B type I (SR-BI), cluster of differentiation 81 (CD81), claudin-1 (CLDN1), and occludin (OCLN). (3) Endocytosis: the interaction of viral envelop proteins with the cell receptors and co-receptors triggers the internalization of the virus into an endosome via endocytosis. (4, 5) Fusion, uncoating, and genome release: once inside the endosome, the virus undergoes fusion with the endosome membrane, leading to the uncoating of the nucleocapsid and the release of the viral genetic material into the cytoplasm. This diagram was created with BioRender.com.

The entry of HCV virion into hepatocytes is mediated by the interaction of the E1/E2 heterodimer with host cell receptors, including cluster of differentiation 81 (CD81), scavenger receptor class B type I (SRB1), claudin 1 (CLDN1), and occludin (OCLN) [32]. The membrane fusion takes place in endosomes at low pH conditions that trigger conformational changes in the E1/E2 heterodimer located at residues 272–285, leading to the release of the genetic material to the cytoplasm. The first step of viral entry is facilitated by the interaction of the E2 HVR1 domain with the SRB1 receptor. This interaction induces conformational changes and exposes the E2 core region and CD81 binding loop. Clathrin-mediated endocytosis is achieved by the interaction of E2 and CD81 (Figure 4). The neutralization of HCV is usually mediated by the action of nAbs developed against the E2 protein. Many antibodies have been identified and developed against the linear and discontinuous regions of the E2 protein, which are known as Ars 1–5, Epitopes I-III, or domains A-E [13]. The crystal structure of the E2 ectodomain in complex with fragments of two different antibodies has been elucidated by two research groups, and the derived information has significantly advanced the understanding of protein structure. The ectodomain structure consists of a central immunoglobulin-like β-sandwich that is stable through conserved disulfide bonds. The N-terminal region consists of a β-strand and a short α-helix, while the C-terminal region consists of antiparallel β-sheets and short α-helices [33,34]. 

The advancement in understanding the structure of the E2 protein has contributed to the identification of antigenic sites and domains crucial for the development of an HCV vaccine able to induce nAbs. The E2 ectodomain from various HCV genotypes has been characterized through complexing with different antibodies, including broadly neutralizing antibodies (bnAbs). The HCV E2 protein is a highly variable protein and widely used for developing bnAbs. Three hypervariable regions have been identified in the E2 protein: HVR1, aa384–409, HVR2, aa460–485 and the igVR, aa570–580. The HVR1 domain has an immunodominant epitope situated at the N-terminal. During virus infection, mutations in this region can lead to the escape of HCV variants from HCV-specific nAbs. HVR1 exhibits higher variability among the different HCV genotypes and subtypes. Other regions of E2 have less variability or are conserved across many genotypes, especially in areas important for the binding of cellular receptors (CD81) during HCV infection. This region has three conserved residues and encompasses three epitopes recognized by bnAb. These epitopes are referred to as Epitope I (aa412–423) situated at the N-terminal, Epitope II (aa428–446) located at the front layer, and Epitope III (aa518–542) situated at the binding loop of CD81. The effectiveness of antibodies against these epitopes has yielded varying results with different outcomes. The E2 protein exhibits three distinct conformations: the hairpin, open, and semi-open conformations. Monoclonal antibodies (mAb) have been developed against the hairpin conformation, including mouse mAbAP33 and mAb24 human mAb HCV1 [35]. The HCV1 and AP33 mAbs have shown broadly neutralizing activities in mice and chimpanzees. The contact residues of both E1 and E2 were shown to be necessary for antibody-mediated virus neutralization [13,20]. The full-length structure of the E1/E2 heterodimer Gt 1a was further investigated by another group. The group has discovered two additional regions in the E2 protein: the base (aa645–700) and the stem (aa 701–717), which are connected with the transmembrane domain by nine disulfide bonds [30]. Recently, Kumar et al. have characterized the interaction of E2 and CD81 receptors by molecular docking model and found that the region with residues 418–422 in E2 undergoes a displacement. Additionally, they also identified that AS412 in E2 is essential for the proper interaction with CD81 [31,35]. Another study has been conducted recently on the vaccine candidate using recombinant HCVcc with a deletion in HVR1. The study observed increased accessibility of nAbs (AR3A and AR4A) with reduced immunogenicity, suggesting the complex role of this region in immunogenicity [36,37]. Thus, during the design and development of the HCV vaccine using immunogens from different regions, it is crucial to consider the immunogenic parts and epitopes of broadly neutralizing antibodies (bNAbs) for higher elicitation of immunogenic responses [22].

### 3.3. Core Proteins

Various vaccine candidates have been designed against HCV by using different strategies and multiple combinations of proteins (structural and non-structural proteins). They have been evaluated for the stimulation of immunogenic responses, but only a few reports have explored the use of Core protein as an antigen for HCV vaccine development [17,38,39,40]. Some recent publications have reported that the vaccine based on a combination of core E1/E2 may induce a better immunogenic response than the virus-like particle assembly and chimeric protein of NS3 epitopes from three HCV genotypes [17,26,41]. Additionally, some HCV vaccines have been developed in transgenic plants. HCV Core protein was expressed in transgenic canola seed, and the extract from these seeds was mixed with an oil adjuvant for mouse vaccination. The vaccination has induced significant humoral (IgG) and Th1-biased immunogenic responses. The cytokine staining has shown that the immunization of mice with the total seed extract induced both CD4+ and CD8+ T cells to release IFN-γ [42]. Another recent study has shown that plasmid DNA carrying the HCV Core sequence was transfected into mesenchymal stem cells (MCS) before the injection of cells into Balb/c mice. The immunized mice demonstrated a superior humoral immunogenic response and produced core-specific antibodies. The transfected MCS-treated mice also exhibited a significant increase in splenocyte proliferation compared to the control group l [43].

### 3.4. Non-Structural Proteins

Some published papers from different research groups have discussed the use of non-structural proteins for HCV vaccine development, either alone or in combination. Among the HCV proteins, the NS3 and Core proteins have shown high conservation and contain many T cell determinants. Various technologies have been employed to study combinations of these proteins, either alone or in multi-tope formulations, aiming to induce Th1-biased immunogenic responses and clear HCV infection. However, the inclusion of an adjuvant in the vaccine formulation has also been shown to be necessary to elicit robust immune responses [19,44]. The heat shock protein (HSP) gp96 has widely been used as a natural adjuvant in viral vaccine formulations to induce innate and adaptive immune responses. In a recent study, the HCV Core-NS3 and Core-NS3-NT (gp96) proteins were expressed in cos-7 cells, and the purified proteins were used for mouse vaccination. A significantly high level of total IgG was observed in vaccinated mice as compared to the control group at weeks 3 and 11. The IFN-production level was high at weeks 3 and 11, but drastic variation was observed for the IL-4 level in the vaccinated and control groups at week 3. Based on the results obtained from different mouse groups, the long-term potency was observed by using the protein/protein + rNT (gp96) vaccine formulation [19]. In another study, it was demonstrated that the immunogenic epitope (1095–1379 aa) of the partial NS3 gene significantly induced the total antibodies and IgG2a, Interferon (IFN)-γ, and Interleukin (IL)-4 [45]. In NS4A and NS3 mice, immunization was performed, which resulted in higher expression levels of NS3 and NS3-specific antibodies and an IgG2a/IgG1 ratio (420 vs. 3) [46]. A DNA vaccine encoding HCV-3a NS3/NS4A was employed for the immunization of C57BL/6 mice, and a significant cell-mediated response was observed [46]. 

### 3.5. Use of Multi-Epitopes

Previous studies have reported that multi-epitope vaccines can elicit varying levels of immune responses. A plasmid DNA containing a combination of peptides and CD8 + T cell epitopes from the Core (132–142), NS3 (1073–1081), and NS5B (2727–2735), along with a ThCD4 + epitope from NS3 (1248–1262) and combined with a B-cell epitope from E2 (412–426) can trigger a higher immune response against HCV. The level of IFN-γ was significantly higher in peptide vaccine adjuvanted with Montanide ISA 720 after three doses of vaccinations as compared to two doses of plasmid and single doses of peptide vaccine. The triple doses of peptide vaccine induced a higher level of IFN-γ/IL-4 ratio as compared to other tested combinations [47]. Another study has indicated that the vaccination of BALB/c mice with a dose of 800 ng to 16 µg of multiple antigenic peptides made by combining conserved epitopes of HCV E1, E2, NS4B, NS5A, and NS5B has produced elevated immunogenic responses and caused an induction of IFN-γ-producing CD4+/CD8+ T-lymphocytes in the immunized mice [15,48].

### 3.6. Contributions of Monoclonal Antibodies to HCV Vaccine Development 

Monoclonal antibodies (mAbs) play an important role in the development of HCV vaccines by aiding in understanding the viral proteins and immune responses during the viral infection. Their significant role in the development of the HCV vaccine can be attributed to various mechanisms, such as: (1) Identification of neutralizing antibodies: Monoclonal antibodies can be derived from individuals who have successfully eliminated the viral infection, enabling researchers to discern and analyze antibodies with the capacity to neutralize the virus. Neutralizing antibodies are valuable targets for the development of vaccines due to their ability to interfere with HCV-infecting liver cells [49]. (2) Epitope mapping: Monoclonal antibodies can locate precise epitopes on viral proteins that neutralizing antibodies specifically recognize. Researchers can identify critical areas of the virus that the immune system can attack by gaining insight regarding monoclonal antibody interactions with viral proteins. The acquisition of this information is crucial to developing vaccine candidates capable of inducing a comparable immune response [50,51]. (3) Vaccine design and evaluation: The efficacy of HCV vaccine candidates can be assessed through the utilization of monoclonal antibodies. Researchers can evaluate the capacity of vaccine-induced antibodies to neutralize various strains or variants of HCV. This procedure aids in determining the potency of the immune response the vaccine elicits, thereby guiding the selection of the most promising vaccine candidates for further development [16]. (4) Passive immunization involves the direct administration of purified antibodies, specifically monoclonal antibodies, to at-risk patients. This strategy offers prompt protection against the virus and can be employed in circumstances where a vaccine has not yet been developed or an insufficient immune reaction to vaccination is exhibited [52,53]. In conclusion, monoclonal antibodies have made a significant contribution to the progress of HCV vaccine development by helping in the identification of viral targets, facilitating the design, testing the vaccines, and opening up new ways to prevent and treat HCV infection.

## 4. Knowledge Obtained from Vaccines Targeting SARS-CoV-2

COVID-19, which is caused by SARS-CoV-2, has emerged as a serious health problem throughout the world, posing difficulties in developing safe and efficient antiviral medicines and preventive vaccines. Vaccine development is a lengthy and complex process that often requires years of research and testing before being approved for human use. However, in 2020, scientists were able to achieve remarkable success in manufacturing safe and effective coronavirus vaccines in record time [54]. This achievement was only made possible due to the collaborative efforts of academia, researchers, and pharmacists, along with financial backing, guided by cumulative knowledge from many years of scientific study. Coronavirus vaccines have been developed in several forms, including inactivated vaccines, nucleic acid vaccines, adenovirus vector-based vaccines, and vaccines containing recombinant components. Researchers have conducted clinical trials on approximately 70 vaccine candidates, with 20 of them having progressed to the final rounds of testing. Over ten of them have been certified for emergency use in a variety of nations worldwide. Among these, two extremely effective mRNA COVID-19 vaccines developed by Pfizer-BioNTech and Moderna have gotten emergency use authorization [55]. 

Dr. Katalin Kariko and her team pioneered the fundamental lessons of mRNA’s therapeutic utility, perhaps most notably in collaboration with Drew Weissman at the University of Pennsylvania (Dr. Katalin Kariko and D. Drew Weissman have won the Nobile Prize for their work on the COVID-19 vaccine in 2023), and this ground-breaking work paved the way for the development of the first mRNA-based therapeutic approved for human use. Several recent publications have highlighted the advancements in the field of mRNA technology and delivery using the lipid nanoparticle (LNP) carrier (the mRNA-LNP) [54,55,56,57]. The mRNA-LNP platform has the potential to fundamentally alter the way diseases are treated. This initiative was sparked by the critical function of mRNA-LNP vaccines in preventing and protecting against COVID-19. Although adverse reactions to these medications have been observed, continuous research to determine the underlying causes would allow for future advancements in mRNA-vaccine LNP’s potential. The establishment of mRNA-LNP as a viable alternative platform for vaccine production paves the way for the eventual eradication of various intractable infectious illnesses. mRNA-based vaccines are appealing because they facilitate and accelerate antigen design for in vivo efficacy evaluation. Additionally, manufacturing expertise needs to operate on a global scale [58]. This is the first time mRNA-based vaccinations have been licensed for human use, and it represents a watershed moment for science and public health [59,60,61]. As discussed previously, mRNA vaccines induce immunological responses by transfecting human cells with synthetic mRNA encoding viral antigens (in this case, spike protein or protein motifs). Once the nucleic acid reaches the cell’s cytosol, the mRNA vaccination drives the cell to manufacture the specific viral antigens encoded by the mRNA [62]. The best significant achievements of these vaccines were: (1) the mRNA modification and purification procedure, which reduced the innate immune response and increased mRNA stability; and (2) effective intracellular delivery, which facilitated cellular uptake of mRNA and protected it from RNase breakdown [55]. These RNA vaccines induce robust antibody responses against the SARS-CoV-2 coronavirus, but they have not been shown to be as effective in stimulating CD8+ T cells as the AstraZeneca/Oxford vaccine (adenoviral vector vaccine). Recent animal studies have indicated that combining an RNA coronavirus vaccine with an adenoviral vector vaccine (the AstraZeneca/Oxford vaccine) may enhance immunological response in mice by rousing CD8+ T cells better than either vaccination alone [63,64]. Clinical trials should be conducted to confirm these early findings. Thus, what can we learn about the remarkable development of the SARS-CoV-2 vaccine? To begin, when there is sufficient interest and resources, the time required to develop and manufacture a vaccine can be greatly decreased. Second, mRNA vaccines are very effective, capable of quick development, and cost-effective to produce. Thirdly, preliminary findings indicate that combining COVID-19 vaccination technology enhances the immune response at the cellular level. Is it therefore conceivable to leverage all of the knowledge gathered from COVID-19 vaccinations to accelerate the development of HCV vaccines? Regrettably, just partly. As noted in the section on difficulties, numerous obstacles remain, as HCV biology and immunology are much different from those of SARS-CoV-2. However, the unexplored option of an HCV mRNA-based vaccine would undoubtedly benefit from the lessons learned and improvements in the field of RNA-based vaccines against SARS-CoV-2 [54].

## 5. The Government’s Role in Ensuring Affordability and Accessibility in the Development of the HCV Vaccination 

The government possesses the ability to provide financial assistance in the form of subsidies for HCV vaccines, making them more economically accessible for both individuals and healthcare systems. One potential strategy to achieve this is to engage in negotiations with vaccine makers to get reduced prices. Another approach could involve offering direct financial assistance to researchers or organizations to help offset the expenses associated with acquiring vaccines [3]. The government can enhance their purchasing power through the implementation of public procurement strategies for HCV vaccinations, as well as leverage their purchasing power by procuring vaccinations in large quantities, facilitating negotiations for more favorable pricing terms, and establishing a reliable and consistent vaccine supply for their respective populations. According to Gianfredi et al. [64], it has been found that governments have the potential to engage in collaborative efforts with pharmaceutical companies and other private sector entities in order to facilitate the development and distribution of HCV vaccines. These collaborations may encompass collaborative research and development endeavors, agreements for the transfer of technology, as well as mutual investments in the production and marketing of vaccines [65,66]. The government can also participate in international alliances such as Gavi, the Vaccine Alliance, or other global initiatives that prioritize the enhancement of vaccine accessibility in countries with low- and middle-income levels. These alliances offer financial assistance, technical knowledge, and collective buying strategies in order to guarantee the accessible and fair availability of vaccines [67]. It is also the government’s ability to incorporate HCV vaccinations into their respective national immunization programs, akin to the inclusion of prevailing vaccines targeting ailments such as hepatitis B, measles, and polio. The incorporation of HCV vaccinations into normal immunization schedules facilitates their availability to a wider demographic. In this manner, focused immunization initiatives can be achieved that specifically cater to populations at heightened risk, such as individuals engaged in drug injection practices (PWID) or those involved in multiple sexual partnerships. These programs have the capacity to offer complimentary or reduced-cost vaccinations in environments that are conveniently accessible to these specific populations, such as harm reduction centers, clinics, or outreach initiatives. The incorporation of HCV vaccination into broad health insurance coverage can be actively pursued and regulated by the government. According to Bush et al. [68], implementing this measure will effectively mitigate the financial burden on persons seeking vaccination, hence enhancing accessibility, especially among those with constrained financial means. Governments have the potential to allocate resources towards public awareness campaigns aimed at educating the general community of the significance of the HCV vaccine as well as the existence of accessible, cost-effective, or complimentary vaccination initiatives. According to Ha and Timmerman [69], this approach has the potential to address the issue of vaccine hesitancy and enhance the demand for HCV vaccines. Governments have the opportunity to consider several strategies aimed at promoting equitable intellectual property rights and facilitating agreements for the transfer of technology. Authorities can explore various approaches to foster fair intellectual property rights and encourage technology transfer agreements. Additionally, by promoting local production or establishing licensing agreements with vaccine manufacturers, they can increase the availability and affordability of HCV vaccinations [70]. It is also possible for the government to engage in partnerships with non-governmental organizations (NGOs) and community-based organizations that operate within the realm of hepatitis C prevention and treatment. These collaborative alliances have the potential to effectively target marginalized communities, enhance knowledge dissemination, and streamline the distribution of HCV vaccinations in places with limited resources [71,72].

The collaboration between scientific, sociopolitical, and economic aspects is vital for the development of HCV vaccines and the management of disease spread, particularly in low- and middle-income countries. This collaborative approach ensures that vaccines are scientifically sound, culturally appropriate, economically feasible, and easily accessible to those who need them the most. By working together, stakeholders can overcome the multifaceted challenges associated with HCV and accelerate progress toward HCV elimination goals, particularly in third-world countries with low and middle-income countries (e.g., Egypt) in which HCV is a serious health problem. This is the reason why we focused this review article on the important role of collaboration between the government and the scientific community.

## 6. Future Prospects and Approaches in the Development of an HCV Vaccine

The following are prospects and approaches to the development of the HCV vaccine.

### 6.1. Innovations and Advancements in HCV Vaccine Development Methods and Technologies

The primary focus of current HCV vaccine options is to stimulate the production of antibodies that can effectively neutralize the virus. However, in the future, scientists may try to make vaccines that activate a wider immune response, including cellular immunity, to effectively eliminate the virus and provide long-term protection [14]. The emergence of innovative vaccine platforms, such as mRNA and viral vector-based technologies, artificial intelligence-based technology, RNAi (miRNAs, siRNAs, and short hairpin RNAs), plant-based vaccines, and nanotechnology-based vaccines (nanoparticles), presents new prospects for the development of HCV vaccines. These platforms have demonstrated promising outcomes in combating other viral diseases and could be explored for their potential in HCV vaccine research [22,23,25,42,73,74]. The genetic variability of HCV poses a challenge in vaccine development. To overcome this, personalized vaccine approaches targeting specific HCV genotypes or subtypes prevalent in various regions may be pursued, allowing tailored protection against local HCV strains [75]. Combining HCV vaccines with other vaccines, such as those targeting hepatitis B virus (HBV), could offer a comprehensive strategy to prevent both hepatitis C and hepatitis B infections. This approach may be particularly valuable for populations at high risk of dual infections [5].

### 6.2. Integration of HCV Vaccines into Public Health Programs

To effectively incorporate HCV vaccines into public health strategies, it is imperative to identify and focus on populations at high risk, such as individuals engaged in injection drug use (PWID), those with multiple sexual partners, and healthcare professionals [76]. To effectively execute vaccination programs, guarantee the availability of vaccines, and assess the impact of HCV immunizations on disease prevalence, it is necessary to foster collaboration among governmental entities, public health agencies, and healthcare practitioners [4]. The efficient integration of HCV vaccinations into public health initiatives will rely on the crucial objective of attaining elevated vaccine coverage rates and proficiently managing apprehensions about affordability and accessibility. Tuckerman et al. [77] have proposed a range of methods that span many approaches. These strategies include the provision of subsidies to mitigate vaccine costs, the expansion of vaccination programs to underserved locations, and the utilization of global coalitions to negotiate vaccine prices.

### 6.3. Collaborative Efforts and Lessons from Global Vaccine Initiatives

The establishment of partnerships between researchers, public health organizations, and pharmaceutical companies would enhance the exchange of knowledge, data, and resources about the development of a vaccine for HCV. The study conducted by Druedahl et al. [78] suggests that valuable insights can be gained from examining the achievements of effective worldwide vaccination campaigns, namely those targeting hepatitis B, human papillomavirus (HPV), and SARS-CoV-2. These successful initiatives can serve as a basis for informing the development strategies of a potential vaccine for HCV. Global partnerships and funding mechanisms, such as those established by international organizations like the World Health Organization (WHO) or Gavi, the Vaccine Alliance, have the potential to significantly contribute to the advancement of HCV vaccine development and promote equitable global access to vaccines [78]. Facilitating the alignment of regulatory standards and processes among nations can enhance the efficiency of worldwide licensing and implementation of HCV vaccinations. According to Druedahl et al. [78], the use of collaborative initiatives can optimize the process of vaccine development, minimize redundant endeavors, and expedite the availability of vaccinations that are both safe and efficacious. Additionally, the implementation of diplomatic initiatives and the fostering of international collaboration can effectively tackle geopolitical obstacles and promote fair allocation of high-cost vaccines against HCV, particularly in low- and middle-income nations where the prevalence of HCV tends to be most pronounced. The success of worldwide initiatives for the development of an HCV vaccine can be enhanced by the implementation of strategies such as resource sharing, knowledge transfer, and capacity building [79].

## 7. General Advancement and Considerations in HCV Vaccine

The current status of vaccine development for HCV involves various initiatives and considerations that are being addressed [13,80]. The researchers want to specifically target and address comprehensive immune responses, with a particular focus on the production of antibodies that can efficiently kill the virus [81,82]. However, it is important for future research to explore more extensive immune responses, such as cellular immunity, to achieve long-lasting immunity and successfully eliminate the virus [83]. The use of novel vaccination platforms, such as mRNA and viral vector-based technologies, has promising promise for the development of a vaccine against HCV [84,85]. The effectiveness of these platforms in managing other viral infections has been well documented, indicating their potential suitability for the production of HCV vaccines. HCV has considerable genetic heterogeneity, posing a substantial challenge that calls for the investigation of customized vaccine approaches targeting certain HCV genotypes or subtypes prevalent in specific geographic regions [86,87]. Moreover, the incorporation of HCV vaccinations alongside other vaccines, such as those specifically targeting the hepatitis B virus, presents a promising approach toward a comprehensive preventive strategy against both hepatitis C and hepatitis B infections [88,89]. This technique has significant potential for patients who are at an elevated risk of getting both disorders concurrently. The present initiatives are focused on advancing the development of effective HCV vaccines and addressing the complexities associated with this infectious disease [90,91]. Future research and collaboration in the development of a vaccine for HCV should prioritize many crucial proposals [92]. Before using antibody-based strategies, it is important to take a close look at and learn more about larger immune responses, such as cellular immunity [14,93]. This has the potential to result in enhanced and enduring protection against HCV. Also, more research needs to be done on new platforms for vaccination, like mRNA and viral vector-based technologies, so that their full potential can be used and they can be changed to help make an HCV vaccine [54,93]. The acceleration of translating these technologies into viable vaccines necessitates collaborative endeavors among researchers, industry stakeholders, and regulatory agencies [94,95,96]. Furthermore, it is imperative to explore individualized vaccine strategies that are specifically designed to target distinct HCV genotypes or subtypes that are widespread in various geographical regions [97,98,99]. 

## 8. Conclusions

In summary, promoting cooperation between manufacturers of HCV vaccines and researchers working on the advancement of vaccines for related viruses like HBV and SARS-CoV-2 holds promise for a comprehensive strategy for preventing both hepatitis C and hepatitis B infections, particularly among populations with increased vulnerability. By implementing these suggestions, future research efforts and collaborations with scientists, academia, politicians, and public health organizations can accelerate the development of effective HCV vaccines and significantly contribute to global initiatives focused on eliminating this significant public health issue.

## Data Availability

Not applicable.
